# Supplementary material for: Qubit teleportation between non-neighbouring nodes in a quantum network
Source: Nature. 2022 May 25;605(7911):663–8. doi: 10.1038/s41586-022-04697-y (PMC9132773; doi:10.1038/s41586-022-04697-y)
Supplement: Supplementary file 1 — This file contains Supplementary Text; equations, figures, tables and references. [file 41586_2022_4697_MOESM1_ESM.pdf]

nature portfolio

https://doi.org/10.1038/s41586-022-04697-y

Supplementary information

Qubit teleportation between non-neighbouring nodes in a quantum network

In the format provided by the authors and unedited

Supplementary Materials for
Qubit teleportation between non-neighboring nodes in a quantum network

S.L.N.Hermans*, M.Pompili*, H.K.C.Beukers, S.Baier, J.Borregaard & R.Hanson†
QuTech and Kavli Institute of Nanoscience, Delft University of Technology
2628 CJ Delft, The Netherlands

*These authors contributed equally to this work
†Corresponding author

Contents

1 Full gate circuit **1**

2 Tailored heralding of the remote entangled states **2**

3 Data acquisition and experimental rates **5**

4 Effect of the 3 key innovations on the teleported state fidelity and experimental rate **6**

5 Estimated fidelity of state to be teleported **6**

1 Full gate circuit

Our quantum network consists of three nodes, Alice, Bob and Charlie. In the experiment, we will teleport a qubit from Charlie to Alice, two non-neighboring nodes. The full gate circuit is shown in Extended Data Figure E4. Prior to the sequence, we do a Charge-Resonance (CR) check on each node to ensure that the communication qubits are in the correct charge state (NV^-) and on resonance with the control lasers. Once all the nodes have passed this check, we do a first round of optical phase stabilization of the interferometers, which enables the entanglement generation using the single click protocol (*1–4*). After these preparation steps, the sequence is triggered on all setups.

On Bob, we initialize the memory qubit into $|0\rangle$ using the communication qubit (*5*). Next, we generate entanglement between the communication qubits of Alice and Bob. When entanglement is heralded, we perform a SWAP operation to store Bob's part of the entangled state on the memory qubit.

We continue with a second round of phase stabilization (not shown in the circuit) and generate entanglement between the communication qubits of Bob and Charlie. Each entanglement attempt slightly decoheres the memory qubit, therefore we limit the number of attempts by a timeout. If we do not succeed within the timeout, we abort the sequence and start over.

During entanglement generation, the memory qubit of Bob picks up an average phase $n\varphi_a$ dependent on the number of entanglement attempts n. Due to the probabilistic nature of the entanglement generation

process, we do not know which attempt will be successful, therefore this phase is unknown at the start of the sequence. To maintain the correct reference frame of the memory qubit this phase needs to be corrected in real-time before any other gate can be applied to the memory qubit. We perform this real-time correction by changing the time between pulses on the communication qubit (*4*). After the phase correction, the decoupling pulse is applied to the memory qubit via the communication qubit. The back-action of this gate causes a Z-rotation on the communication qubit. To rephase the memory qubit, we wait for the same amount of time as it took to herald the second entangled state while decoupling the communication qubit. This imprints a phase $q\varphi_b$ on the memory qubit, which we compensate in an analogous way.

Bob now shares two entangled states; his memory qubit is entangled with Alice and his communication qubit with Charlie. To establish an entangled state between Alice and Charlie we perform a Bell-state measurement on the two qubits of Bob. To do so, we entangle the communication and memory qubits and do a measurement on the communication qubit. We map its state onto the communication qubit and measure the communication qubit. In the basis-alternating repetitive readout, we repeat the measurement sequence twice. During the first readout we map the $|0\rangle$ state to the $|0\rangle$ state of the communication qubit, and in the second readout we map $|1\rangle$ to $|0\rangle$. The first outcome is used to assign the state and the second outcomes serves as a check. By continuing the sequence only when we measure consistent patterns (for instance $(m1, m2) = (1, 0)$) we increase our average readout fidelity. After the readout procedure, we perform a CR check on Bob to filter out any event where Bob was in the wrong charge state.

Bob communicates to Charlie which gate operation should be done to obtain the correct entangled state. Which operation is required is determined by the outcomes of the Bell-state measurement on Bob and by which detector heralded the individual links. Charlie performs the feed-forward gate operation and subsequently stores its part of the entangled state on the memory qubit using a SWAP gate. At this point in the sequence the teleporter is ready.

To prepare the state that is to be teleported, we initialize the communication qubit at Charlie and perform the desired qubit rotation.

To teleport the qubit, we perform a Bell-state measurement on the qubits of Charlie. Locally, we entangle the communication qubit with the memory qubit. We readout the communication qubit and use the basis-alternating repetitive readout for the memory qubit. Additionally, we do a CR check on Charlie. Charlie communicates the results of the Bell-state measurement to Alice, and Alice performs a feed-forward operation to obtain the teleported state.

To verify the teleported state, we measure the state of Alice in the corresponding basis. To prevent any bias in the tomography we measure in both directions, e.g. when we teleport $|+Z\rangle$ we measure both along +Z and -Z axes.

2 Tailored heralding of the remote entangled states

In the main text we describe several noise mechanisms that reduce the remote two-node entangled state fidelity. Two of these noise mechanisms, double $|0\rangle$ occupancy and double optical excitation, are accompanied by the emission of an extra photon. This extra photon can be detected using the local phonon-side band (PSB) detectors. By monitoring the PSB detectors, we can real-time reject false heralding events.

In Extended Data Figures E1 and E2, we plot the histograms of the detection times of the PSB photons conditioned on a simultaneous heralding (zero-phonon line, ZPL) photon detection in the same entanglement generation attempt, for the Alice-Bob and Bob-Charlie entangled link respectively. The correlations are measured in the computational (or Z) basis, and in the X and Y basis. In the computational basis we see the behavior dependent on the detection time of the PSB photon as described in the main text together with the simulations (gray bars). In the X and Y basis, all outcomes are equally probable, and the quantum correlations

are washed out.

From the data collected, we can extract the probability to detect these additionally emitted PSB photons. We assume the dark counts of the detectors to be negligible, the PSB detections during the pulse to be fully dominated by the double optical excitation error, and the PSB detections after the pulse to be only caused by double $|0\rangle$ occupancy. By correcting for the PSB detection efficiency, we can estimate the probability for double $|0\rangle$ occupancy and double optical excitation errors. The results are given in Table S1. The double $|0\rangle$ state error is expected to occur with probability α. The extracted numbers correspond well to the parameter values we use during remote entanglement generation ($\alpha_{\text{Alice}} = 0.07, \alpha_{\text{Bob}} = 0.05, \alpha_{\text{Charlie}} = 0.10$ (*4*)). The probability for the double optical excitation to occur depends on the shape and the amplitude of the optical excitation pulse, and differs per node.

2.1 Numerical model

We compare our PSB detection data (previous section) to a numerical model. We model the NV center as a three level system with two stable ground states $|0\rangle, |1\rangle$ and one excited state $|e\rangle$. The optical $|0\rangle \leftrightarrow |e\rangle$ transition is driven by a resonant laser pulse and is assumed to be a closed transition. The Hamiltonian describing the dynamics of the system in a suitable rotating frame is

$$\hat{H} = \Omega(t) |e\rangle \langle 0| + \Omega^*(t) |0\rangle \langle e| , \tag{1}$$

where $\Omega(t)$ describes the (time-dependent) driving of the optical transition. From the excited state, the NV can spontaneously emit a photon and decay to $|0\rangle$. Without specifying the particular mode this photon is emitted in, we simply model such an emission with a Lindblad jump operator of the form $\hat{L}_1 = \sqrt{\gamma} |0, 1_p\rangle \langle e|$. Here γ is the rate of spontaneous emission, $|0, 1_p\rangle$ denotes the state where the NV is in state $|0\rangle$ and one photon was emitted, and we use the convention that when not explicitly stated, there is no emitted photon i.e. $|e\rangle$ denotes the NV in state $|e\rangle$ with zero emitted photons.

To account for double emission errors in the entanglement scheme, we expand the model by letting states $|0, 1_p\rangle, |e, 1_p\rangle$ be coupled by a similar Hamiltonian as in Eq. (1) with the same coupling $\Omega(t)$. Double emission is then captured by a Lindblad jump operator $\hat{L}_2 = \sqrt{\gamma} |0, 2_p\rangle \langle 1_p, e|$. For the specific excitation pulses used in the experiment, we can then numerically solve the Master equation of the system in a basis of $\{|0\rangle, |e\rangle, |0, 1_p\rangle, |e, 1_p\rangle, |0, 2_p\rangle\}$ to obtain the probability of zero (P_0), one (P_1), or two (P_2) photons being emitted from the system ($P_0 + P_1 + P_2 = 1$). Note that in this model, we neglect the probability of emitting more than two photons from the NV.

Assuming an initial state $\sqrt{\alpha} |0\rangle + \sqrt{1-\alpha} |1\rangle$ of the NV center, the state after the optical excitation is then modeled as

$$|\psi\rangle = \sqrt{\alpha} \left(\sqrt{P_0} |0\rangle + \sqrt{P_1} |0, 1_p\rangle + \sqrt{P_2} |0, 2_p\rangle \right) + \sqrt{1-\alpha} |1\rangle . \tag{2}$$

The emitted photons are either PSB ($= 97\%$) or ZPL ($= 3\%$) photons. We model this by performing a standard beam splitter transformation on the photonic modes. Letting $\hat{a}^\dagger$ be the creation operator of a photon ($|1_p\rangle = \hat{a}^\dagger |0_p\rangle$), we make the transformation $\hat{a}^\dagger \rightarrow \sqrt{P_z} \hat{a}_z^\dagger + \sqrt{1-P_z} \hat{a}_b^\dagger$, where $\hat{a}_z^\dagger$ ($\hat{a}_b^\dagger$) is the creation operator of a ZPL (PSB) photon and $P_z = 3\%$. Consequently, $|1_p\rangle \rightarrow \sqrt{P_z} |1_z\rangle + \sqrt{1-P_z} |1_b\rangle$, where $|1_z\rangle$ ($|1_b\rangle$) is an emitted ZPL (PSB) photon.

The photons can be emitted either inside or outside the detection time window, i.e. the time interval in which detected photons are accepted. This time interval is in general different for the PSB and ZPL photons.

This results in the following transformations:

$$\begin{aligned}
\left|1_z\right\rangle &\rightarrow \sqrt{P_{dz,1}}\left|1_{d,z}\right\rangle + \sqrt{1-P_{dz,1}}\left|1_{nd,z}\right\rangle &(3)\\
\left|1_b\right\rangle &\rightarrow \sqrt{P_{db,1}}\left|1_{d,b}\right\rangle + \sqrt{1-P_{db,1}}\left|1_{nd,b}\right\rangle &(4)\\
\left|2_z\right\rangle &\rightarrow \sqrt{P_{dz,2}}\left|2_{d,z}\right\rangle + \sqrt{P_{dz,3}}\left|1_{d,z}\right\rangle\left|1_{nd,z}\right\rangle + \sqrt{1-P_{dz,2}-P_{dz,3}}\left|2_{nd,z}\right\rangle &(5)\\
\left|2_b\right\rangle &\rightarrow \sqrt{P_{db,2}}\left|2_{d,b}\right\rangle + \sqrt{P_{db,3}}\left|1_{d,b}\right\rangle\left|1_{nd,b}\right\rangle + \sqrt{1-P_{db,2}-P_{db,3}}\left|2_{nd,b}\right\rangle &(6)\\
\left|1_z\right\rangle\left|1_b\right\rangle &\rightarrow \sqrt{P_{dzb,1}}\left|1_{d,z}\right\rangle\left|1_{d,b}\right\rangle + \sqrt{P_{dzb,2}}\left|1_{nd,z}\right\rangle\left|1_{d,b}\right\rangle &(7)\\
&\quad + \sqrt{P_{dzb,3}}\left|1_{d,z}\right\rangle\left|1_{nd,b}\right\rangle + \sqrt{1-P_{dz,2}-P_{dz,2}-P_{dz,3}}\left|1_{nd,z}\right\rangle\left|1_{nd,b}\right\rangle .
\end{aligned}$$

The probabilities $P_{dz,1}, P_{db,1}, \ldots$ are defined in table S2 and are found through the numerical simulation described above.

Finally, we model transmission loss with standard beam splitter transformations acting on the photon modes emitted in the detection window. Letting $\hat{a}^\dagger_{d,z}$ ($\hat{a}^\dagger_{d,b}$) be the creation operator of a ZPL (PSB) photon emitted in the detection time window, we make the transformations

$$\hat{a}^\dagger_{\mathrm{d,z}} \rightarrow \sqrt{\eta_z}\hat{a}^\dagger_{\mathrm{d,z}} + \sqrt{1-\eta_z}\hat{a}^\dagger_{\mathrm{nd,z}} \tag{8}$$

$$\hat{a}^\dagger_{\mathrm{d,z}} \rightarrow \sqrt{\eta_b}\hat{a}^\dagger_{\mathrm{d,b}} + \sqrt{1-\eta_z}\hat{a}^\dagger_{\mathrm{nd,b}}. \tag{9}$$

where η_z is the total transmission efficiency from the NV to the central beam splitter while η_b is the total transmission and detection efficiency of the PSB photons. The operators $\hat{a}^\dagger_{\mathrm{nd,z}}$ and $\hat{a}^\dagger_{\mathrm{nd,b}}$ describe the lost/undetected modes. Tracing over the undetected modes, the output state of a single NV can be written as

$$\rho_\psi = \rho_0 \otimes \left|0\right\rangle\left\langle 0\right|_{\mathrm{d,b}} + \rho_1 \otimes \left|1\right\rangle\left\langle 1\right|_{\mathrm{d,b}} + \rho_2 \otimes \left|2\right\rangle\left\langle 2\right|_{\mathrm{d,b}} , \tag{10}$$

where we have neglected any coherence between the photonic PSB modes since these are accompanied by undetected non-radiative decay (phonon emission). The unnormalized density matrices ρ_0, ρ_1, and ρ_2 describe the state of the NV center communication qubit and the ZPL photons emitted in the time window of the ZPL detectors and transmitted to the central beam splitter. In the limit $\eta_z \ll 1$, we can neglect terms of $\left|2_{\mathrm{d,z}}\right\rangle$ and these density matrices will all be of the form

$$\rho_j = \sum_{i=1}^{4} \left|\phi_{i,j}\right\rangle\left\langle\phi_{i,j}\right| , \tag{11}$$

where $\left|\phi_{i,j}\right\rangle = (a_{i,j}\left|1\right\rangle + b_{i,j}\left|0\right\rangle)\left|0_z\right\rangle + c_{i,j}\left|0\right\rangle\left|1_{\mathrm{d,z}}\right\rangle$ and $j = 0, 1, 2$. In Eq. (11) i refers to the different number of lost undetected photons

$i = 1$, zero photons being lost

$i = 2$, one ZPL photon being lost

$i = 3$, one PSB photon being lost

$i = 4$, two photons being lost, either two ZPL, two PSB or one ZPL and one PSB

and j to the number of detected PSB photons. We note that all $a_{i,1}$ and $a_{i,2}$ will be zero since ρ_1 and ρ_2 are accompanied by PSB photons (see Eq. (10)) meaning that the NV was in state $\left|0\right\rangle$. Furthermore, the only non-zero term in ρ_2 will be $b_{i,2}$ since two PSB photons were emitted, meaning that no ZPL photon was emitted since we neglect higher order emissions.

The only term in Eq. (10) from which remote spin-spin entanglement between two NVs can be created is $\rho_0 \otimes |0\rangle\langle 0|_{\text{d,b}}$ since this does not have any detected PSB photons. However, PSB and ZPL photons that were emitted but not detected will still decrease the entangled state fidelity. Such events are responsible for the contributions of $|\phi_{2,0}\rangle$, $|\phi_{3,0}\rangle$ and $|\phi_{4,0}\rangle$ in ρ_0. The only term where no PSB photons were emitted and no ZPL photons were undetected is $|\phi_{1,0}\rangle = \sqrt{1-\alpha}\,|1\rangle\,|0_{\text{zpl}}\rangle + \sqrt{\alpha}\,|0\rangle\,(\sqrt{P_0}\,|0_{\text{zpl}}\rangle + \sqrt{P_1 P_{\text{zpl}} P_{\text{d,zpl}}}\,|1_{\text{d,zpl}}\rangle)$.

The combined state from the two NV centers before the central beam splitter is $\rho_\psi \otimes \tilde{\rho}_\psi$, where $\tilde{\rho}_\psi$ (the state of the second NV) is of the same form as in Eq.10 but including that parameters such as initial rotation (α), driving strength (Ω) and transmission efficiencies (η_z, η_b) can be different for the two centers. Furthermore, we include a phase difference between the two paths to the central beam splitter. The central beam splitter is modeled as a perfect 50:50 beam splitter and the finite detection efficiency of the output detectors is assumed to be equal and can be directly included in the transmission efficiencies (η_z) while dark counts are negligible in the experiment and not included. Finally, we include non-perfect visibility between the ZPL photons by reducing the coherence between the output modes of the beam splitter by a factor v. This visibility is estimated from experimental data and can e.g. originate from slightly off-resonant driving of the NV centers.

3 Data acquisition and experimental rates

At multiple points during the experimental sequence we make a decision on whether to continue the protocol or not. For example, after successful heralding of a two-node entangled state, we can decide to abort the protocol based on whether the flag was raised by any detection of a PSB photon. As all these signals come in real-time, these decisions can be made in real-time, and the sequence can be aborted whenever appropriate. However, we choose to postpone these decisions to the processing after the data acquisition and continue the sequence in any case. In this way, we gain more insight in the performance of the experiment, at the expense of just a marginal increase in experimental time. Processing steps taken after the data acquisition:

- More precise temporal selection than 15 ns.
- PSB rejection.
- Selection of readout outcomes during the Bell-state measurements, including selection on consistent readout patterns for the memory qubit readout.
- Selection on successful Charge-Resonance checks during the sequence.
- Ensure that the last optical phase measurement (before feedback) prior to the heralding event is below $< 50^o$.
- High enough photon count rates on Alice during qubit initialization and optical pulsing, averaged over the second before the heralding signal comes in. (On Alice we perform gate tuning to keep the qubit on resonance with the control lasers. The gate tuning, in combination with the high repetition rate of entanglement attempts, makes the qubit spectrally jumpy. The control loops during the charge-resonance checks should ensure the resonance condition (*6*), but we use this live-tracking of the photon statistics as an extra check.)

To emphasize, all these processing steps can also be done real-time during the experiment. For the data acquisition, we interleave blocks of measurements with calibrations. The calibrations also serve as an independent measure of the performance of the setups.

We collect the data in blocks of 200 “raw” data points (taking roughly an hour), which result on average in about 30 data points per block after applying the processing steps. We analyzed the data only after completing all data acquisition. Prior to the measurement, we decided on the target total number of data points, the experimental settings, and the processing steps afterwards. The plan was to run a sufficient number of blocks (estimated at 80) such that after processing we would remain with ¿2,800 data points, and at least 450 per cardinal axis. These target numbers were a trade-off between measurement time and expected violation of the classical bound for the conditional teleportation. Unfortunately, after 80% of the data points were acquired, the setups consistently failed two of the calibrations steps due to the formation of ice on Charlie’s diamond sample (the origin of this, either a leak or an outgassing element, is under investigation at time of writing). Therefore, we decided to end the data acquisition, include all data taken up to that point and analyze. In total, we have acquired 79 blocks of data, and we measured 2272 events ($|+X\rangle$ 382, $|-X\rangle$ 385, $|+Y\rangle$ 385, $|-Y\rangle$ 378, $|+Z\rangle$ 375, $|-Z\rangle$ 367) for the conditional teleportation over a time span of 21 days.

We can determine the experimental rate including all overhead (such as CR checks, communication time and phase stabilization) by dividing the number of measured data points by the total measurement time. In Extended Data Figure E5 we plot the experimental rate for both the conditional and unconditional teleportation sequence. In the case of the unconditional teleportation, we accept all Bell-state measurement outcomes on Charlie and therefore the experimental rate is higher. For shorter detection windows during the two-node entanglement, the success probability per attempt is smaller and thus the experimental rate is lower.

4 Effect of the 3 key innovations on the teleported state fidelity and experimental rate

We assess the effect of each innovation on the teleportation protocol. First, we estimate the average state fidelity and experimental rate with a set of baseline parameters based on the performance in (*4*). We use a timeout of 1000 entanglement attempts for the second link (between Bob and Charlie) before aborting the protocol and starting over. In both Bell-state measurements, we continue the sequence for the outcomes ”00” and ”01” (communication qubit, memory qubit), or abort and start over (in the case of conditional teleportation). Then we incrementally add (1) the basis-alternating repetitive readout scheme for the memory qubits,(2) the improved memory qubit coherence and (3) the tailored heralding scheme of the remote entanglement generation. The results are summarized in S3.

5 Estimated fidelity of state to be teleported

The state to be teleported is prepared on the communication qubit of Charlie. Errors in the preparation originate from imperfect initialization and imperfect MW pulses, which are estimated to be $p_{init} = 1.2 \times 10^{-3}$ and $p_{MW} = 8 \times 10^{-3}$ (*7*). Averaged over the six cardinal states, we estimate the state preparation fidelity to be ≈ 0.995.

References

1. C. Cabrillo, J. I. Cirac, P. García-Fernández, and P. Zoller, "Creation of entangled states of distant atoms by interference," *Physical Review A*, vol. 59, pp. 1025–1033, Feb. 1999.

2. S. Bose, P. L. Knight, M. B. Plenio, and V. Vedral, "Proposal for Teleportation of an Atomic State via Cavity Decay," *Physical Review Letters*, vol. 83, pp. 5158–5161, Dec. 1999.

3. P. C. Humphreys, N. Kalb, J. P. Morits, R. N. Schouten, R. F. Vermeulen, D. J. Twitchen, M. Markham, and R. Hanson, "Deterministic delivery of remote entanglement on a quantum network," *Nature*, vol. 558, no. 7709, pp. 268–273, 2018.

4. M. Pompili, S. L. Hermans, S. Baier, H. K. Beukers, P. C. Humphreys, R. N. Schouten, R. F. Vermeulen, M. J. Tiggelman, L. dos Santos Martins, B. Dirkse, S. Wehner, and R. Hanson, "Realization of a multinode quantum network of remote solid-state qubits," *Science*, vol. 372, no. 6539, pp. 259–264, 2021.

5. J. Cramer, N. Kalb, M. A. Rol, B. Hensen, M. S. Blok, M. Markham, D. J. Twitchen, R. Hanson, and T. H. Taminiau, "Repeated quantum error correction on a continuously encoded qubit by real-time feedback," *Nature Communications*, vol. 7, p. 11526, May 2016.

6. B. Hensen, H. Bernien, A. E. Dreaú, A. Reiserer, N. Kalb, M. S. Blok, J. Ruitenberg, R. F. Vermeulen, R. N. Schouten, C. Abellán, W. Amaya, V. Pruneri, M. W. Mitchell, M. Markham, D. J. Twitchen, D. Elkouss, S. Wehner, T. H. Taminiau, and R. Hanson, "Loophole-free Bell inequality violation using electron spins separated by 1.3 kilometres," *Nature*, vol. 526, no. 7575, pp. 682–686, 2015.

7. N. Kalb, P. C. Humphreys, J. J. Slim, and R. Hanson, "Dephasing mechanisms of diamond-based nuclear-spin memories for quantum networks," *Physical Review A*, vol. 97, no. 6, pp. 1–10, 2018.

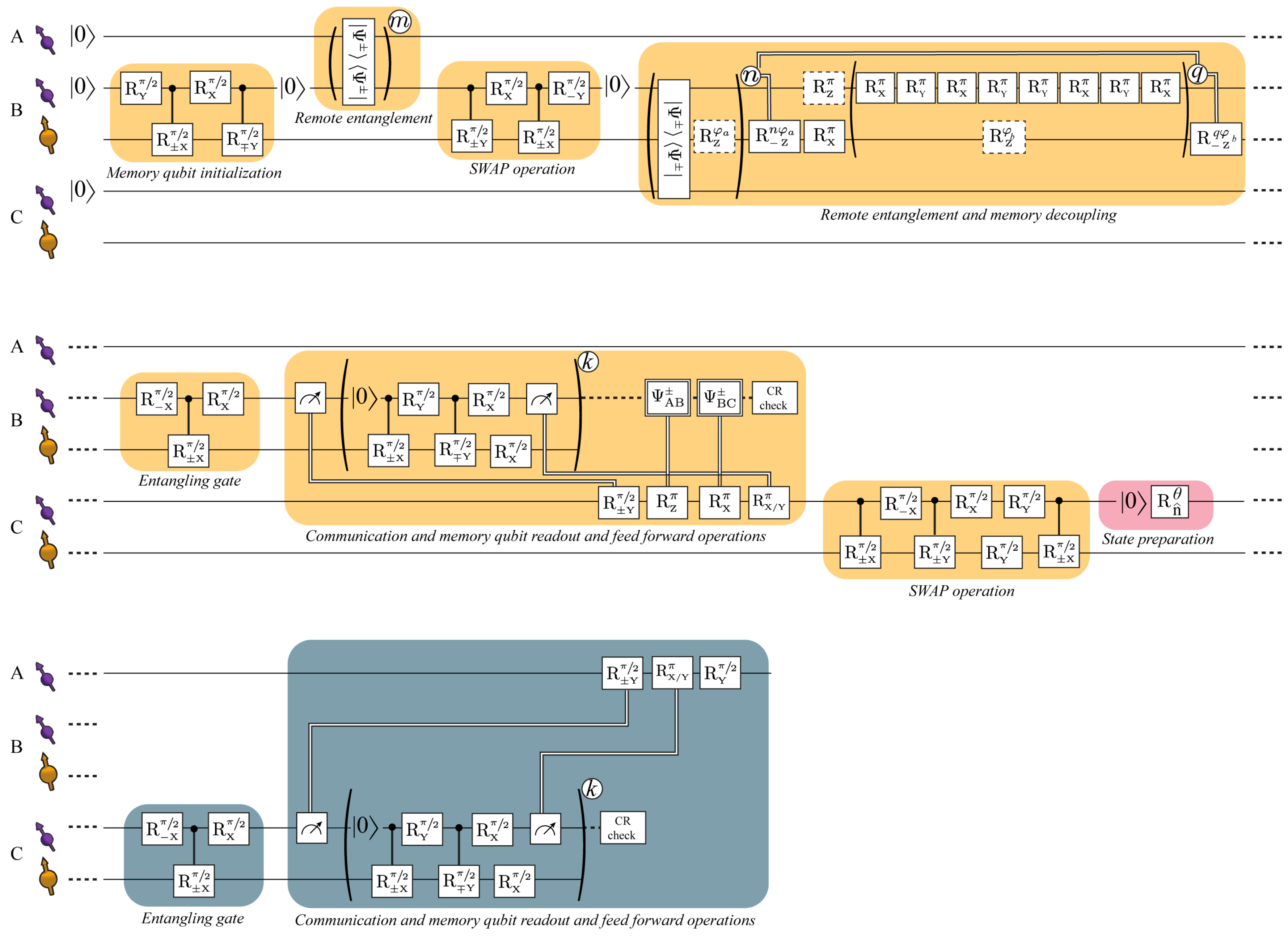

Figure S1: Full gate circuit for the teleportation protocol, see text for the description of each element.

Table S1: Estimated probabilities for the double optical excitation error and the double $|0\rangle$ occupancy error per node (values in percent). All error bars represent one standard deviation.

Node	Double optical excitation probability	Double $|0\rangle$ occupancy probability
Alice	4.1 ± 0.5	7.6 ± 0.4
Bob (with Alice)	2.6 ± 0.3	4.9 ± 0.3
Bob (with Charlie)	6.9 ± 0.8	4.7 ± 0.8
Charlie	5.7 ± 0.4	9.4 ± 0.4

Parameter	Description
γ	Spontaneous emission rate of the excited state.
$\Omega(t)$	Optical driving strength.
α	Initial population of the $|0\rangle$ state.
P_0	Probability of emitting 0 photons (ZPL or PSB).
P_1	Probability of emitting 1 photons (ZPL or PSB).
P_2	Probability of emitting 2 photons (ZPL or PSB or both).
P_z	Probability that an emitted photon is a ZPL photon.
$P_{dz,1}$	Probability that a ZPL photon is within the ZPL detection window, conditioned on a single ZPL photon being emitted.
$P_{db,1}$	Probability that a PSB photon is within the PSB detection window, conditioned on a single PSB photon being emitted.
$P_{dz,2}$	Probability that 2 ZPL photons are within the ZPL detection window, conditioned on two ZPL photons being emitted.
$P_{dz,3}$	Probability that one ZPL photons is within the ZPL detection window and one is not, conditioned on two ZPL photons being emitted.
$P_{db,2}$	Probability that 2 PSB photons are within the PSB detection window, conditioned on two PSB photon being emitted.
$P_{dz,3}$	Probability that one PSB photons is within the PSB detection window and one is not, conditioned on two PSB photons being emitted.
$P_{dzb,1}$	Probability that a ZPL photon is within the ZPL detection window and a PSB photon is within the PSB detection window, conditioned on one ZPL and one PSB photon being emitted.
$P_{dzb,2}$	Probability that a ZPL photon is not within the ZPL detection window and a PSB photon is within the PSB detection window, conditioned on one ZPL and one PSB photon being emitted.
$P_{dzb,3}$	Probability that a ZPL photon is within the ZPL detection window and a PSB photon is not within the PSB detection window, conditioned on one ZPL and one PSB photon being emitte.
η_z	Total transmission and detection efficiency of ZPL photons.
η_p	Total transmission and detection efficiency of PSB photons.

Table S2: Explanation of the parameters used in the numerical simulation of the entanglement generation protocol.

Table S3: Simulated effect of the innovations on the teleported state fidelity and experimental rate.

	Fidelity	Rate (Hz)
Baseline parameters using timeout = 1000, BSM outcomes (communication qubit, memory qubit) = ”00” or ”01”	0.666	1/(53s)
With basis-alternating repetitive readout	0.679	1/(73s)
With improved memory coherence	0.687	1/(73s)
With tailored heralding scheme	0.695	1/(74s)
